# A Study of the Properties of Scaffolds for Bone Regeneration Modified with Gel-like Coatings of Chitosan and Folic Acid

**DOI:** 10.3390/gels9100773

**Published:** 2023-09-23

**Authors:** Aleksandra Bandzerewicz, Kamila Godzieba, Kamil Wierzchowski, Maciej Pilarek, Agnieszka Gadomska-Gajadhur

**Affiliations:** 1 Faculty of Chemistry, Warsaw University of Technology, 00-664 Warsaw, Poland; aleksandra.bandzerewicz.dokt@pw.edu.pl (A.B.);; 2 Faculty of Chemical and Process Engineering, Warsaw University of Technology, 00-645 Warsaw, Polandmaciej.pilarek@pw.edu.pl (M.P.)

**Keywords:** tissue engineering, scaffold, polylactide, chitosan, folic acid, bone regeneration, polymer gelling, gel coatings

## Abstract

The research has been conducted to obtain scaffolds for cancellous bone regeneration. Polylactide scaffolds were made by the phase inversion method with a freeze-extraction variant, including gelling polylactide in its non-solvent. Substitutes made of polylactide are hydrophobic, which limits cell adhesion. For this reason, the scaffolds were modified using chitosan and folic acid by forming gel-like coatings on the surface. The modification aimed to improve the material’s surface properties and increase cell adhesion. Analyses of obtained scaffolds confirmed the effectiveness of performed changes. The presence of chitosan and folic acid was confirmed in the modified scaffolds, while all scaffolds retained high open porosity, which is essential for proper cell growth inside the scaffold and the free flow of nutrients. Hydrostatic weighing showed that the scaffolds have high mass absorbability, allowing them to be saturated with biological fluids. There were also cytotoxicity tests performed on 24 h extracts of the materials obtained, which indicated a lack of cytotoxic effect.

## 1. Introduction

The growing rate of tissue and organ damage is undoubtedly a severe problem affecting an increasing number of people. Until now, transplants have been used to treat them, which allows partial or complete replacement of the damaged organs. Transplantation is an easy and effective method; however, there are some drawbacks. These include the possibility of an adverse immune reaction in the recipient’s body and an increased risk of infection. That is why researchers seek modern and alternative solutions that will allow complete damage reconstruction in a shorter time without complications. Tissue engineering creates excellent opportunities in this area. It is an interdisciplinary field of science that develops materials that entirely replace damaged tissues or improve their functions [1,2].

One of the main goals of tissue engineering is to obtain three-dimensional and porous structures called scaffolds. They are the site of cell attachment, growth and differentiation. Scaffolds should be biocompatible, biodegradable and have a suitable spatial structure with high open porosity [3,4]. Currently, polymeric materials are most commonly used to manufacture such implants. They can be divided into two subgroups: natural polymers (e.g., hyaluronic acid, alginate, chitosan) and synthetic polymers (e.g., polylactide, poly (glycolic acid)). Appropriate mechanical properties, biocompatibility and biodegradability characterise polymeric materials for use in tissue engineering. They also often exhibit high bioactivity and stimulate the adhesion and proliferation of cultured cells [4,5,6].

A particularly common polymer for this type of application is polylactide (PLA), which has been thoroughly studied and successfully applied for many years [7,8,9,10]. Nonetheless, polylactide exhibits some properties that may limit cell adhesion and growth. The –CH_3_ group present in the PLA chain increases the hydrophobicity of the material and reduces the rate of degradation in aqueous environments. Various modifications are made to improve the surface properties of PLA-based materials [11]. One of the most common approaches is applying biomimetic coatings [12,13,14,15]. For this purpose, chitosan (CS) or folic acid (FA) can be used.

Due to the presence of –NH_2_ groups, which undergo protonation, chitosan forms a positively charged chain, promoting cell adhesion and proliferation. Additionally, it can bind to the anions present in the bacterial cell wall, blocking it and interfering with biosynthesis and nutrient transport [16]. Chitosan exhibits anti-tumour effects by promoting the natural death processes of cancer cells through enzymatic, anti-angiogenic and apoptotic mechanisms [17]. Chitosan is used in wound dressings for faster wound scarring by activating and moderating the function of inflammatory cells (neutrophils, macrophages, fibroblasts). Chitosan also has haemostatic properties. Due to its cationic nature, it can bind negatively charged red blood cells, aiding the blood clotting process. Chitosan promotes the adhesion and proliferation of osteogenic and mesenchymal stem cells. Osteogenic cells growing on the scaffold secrete an extracellular matrix, which is then mineralised and transformed into bone tissue. The polymer also promotes the osteogenic differentiation of mesenchymal stem cells [16,17,18,19].

Folic acid, also known as vitamin B9, is an essential compound for the proper functioning of the human body. It is practically insoluble in water and most organic solvents but can dissolve in dilute acids and alkaline solutions. Upon oral administration, its derivatives are immediately distributed throughout all body tissues. Folic acid exhibits an extensive affinity to plasma proteins. In the body, folic acid is biologically active as tetrahydrofolic acid. It is involved in many biochemical processes, including protein and nucleic acid synthesis and amino acid metabolism. Tetrahydrofolic acid is also necessary to form the myelin sheath on nerve fibres. FA in the form of metal salts (calcium or strontium) increases the viability of human osteoblasts. In addition, folic acid salts promote increased bone alkaline phosphatase activity, which determines normal bone development. Metal complexes with FA also promote bone cell replication [20,21,22,23].

Combining these three biomaterials, polylactide, chitosan and folic acid, should result in scaffolds with suitable mechanical and structural properties for bone tissue regeneration while showing improved surface properties to promote cell adhesion and growth. This article describes successful chitosan and folic acid modifications made to a particular type of bone implant, which, to our knowledge, has not been reported yet. It is preliminary research aiming to confirm that such a process has no adverse effect on the scaffolds’ basic mechanical properties and morphology. It is a good introduction to further, more detailed study.

## 2. Results and Discussion

The research was conducted to obtain poly-L-lactide cellular scaffolds modified with chitosan and folic acid, which could be used for cancellous bone regeneration. The properties of modified and unmodified polylactide scaffolds were investigated for comparison.

### 2.1. Preparation of Polylactide Scaffolds and Their Modification with Chitosan and Folic Acid

Scaffolds made of poly-L-lactide were produced using a phase inversion technique with a freeze-extraction variant. The procedure includes a gelling bath phase. This allows a porous structure that mimics the natural structure of spongy bone by gelling the polylactide in its non-solvent. The scaffolds were obtained in a cylindrical form with a diameter of 2 cm and a height of 3 cm. Water was used as the porophore due to its non-toxicity, widespread availability and ease of removal. In order to improve the surface properties, the scaffolds were modified via an immersion method using chitosan and, subsequently, folic acid solutions.

**Figure 1:**
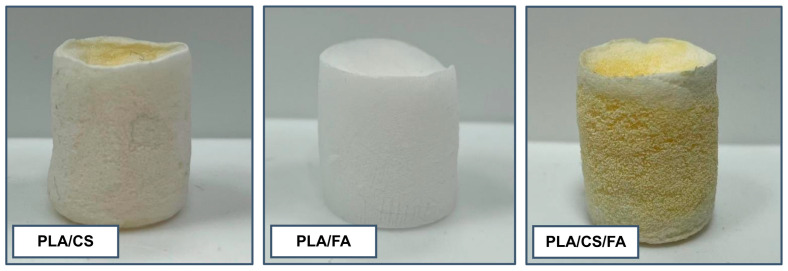
Visual comparison between polylactide scaffolds differing in surface modification type.

The modification was carried out by applying a high-viscosity coating solution and then precipitating a gel-like coating on the surface of the base scaffold. Three types of specimens were prepared: (1) chitosan-modified polylactide scaffolds (PLA/CS), (2) folic acid-modified polylactide scaffolds (PLA/FA) and (3) chitosan- and folic acid-modified polylactide scaffolds (PLA/CS/FA) (Figure 1).

Both the chitosan solution and the folic acid solution were coloured, with the former being slightly yellow and the latter being intensely orange. Non-modified scaffolds are white. Therefore, the effectiveness of the modifications could be assessed organoleptically first. It is apparent that in the case of PLA/CS and PLA/CS/FA scaffolds, the process has been successful. PLA/FA scaffold is only slightly yellowish, which is not clearly visible in the photo, unfortunately. All specimens retained their cylindrical form. The observed changes were reflected in weight gain, respectively, with an average of 2% for PLA/FA and 40% for PLA/CS compared to non-modified scaffolds and 14% for PLA/CS/FA compared to PLA/CS scaffolds. The introduction of chitosan gel layers onto the surface of the samples is, therefore, relatively easy, which is probably due to the appropriate viscosity of the solution used, simultaneously enabling the successive application of the immersion method (supported by the use of reduced pressure) while preventing rapid liquid flow-down from the sample surface. Due to it being less viscous, it is more difficult to coat the surface effectively with the folic acid solution. However, a significant improvement is seen for PLA/CS/FA, indicating that the folic acid probably binds to the chitosan previously incorporated on the surface by weak bonding interactions (hydrogen bonds and van der Waals forces), which does not occur in the case of PLA/FA. As a result, a gel-like structure is formed on the surface of the scaffold in which the chitosan chains are doped with folic acid.

### 2.2. Analysis of Selected Properties of the Scaffolds

The prepared scaffolds were examined for chemical composition, pore structure, open porosity, mass absorbability and compressive strength.

#### 2.2.1. Chemical Composition

The aim of the modification was to enrich the polylactide scaffolds with nitrogen, which should condition better cell adhesion and proliferation on the scaffolds. The effectiveness of the modification process was assessed by elemental analysis (Table 1).

The non-modified scaffolds, made only from polylactide, do not contain nitrogen, so the presence of nitrogen in the other samples is solely due to the modification, the chitosan coating and the enrichment with folic acid, as both substances contain nitrogen in the molecule. The increase in the percentage of nitrogen between PLA/CS and PLA/CS/FA results from the modification with folic acid effectively carried out. In the case of PLA/FA scaffolds, folic acid was embedded in a small amount. The noticeably higher standard deviation of the results for scaffold samples modified with both coatings may indicate a more significant inhomogeneity of folic acid distribution in the scaffold volume than in the case of the chitosan coating. Regardless, the process should be considered adequate.

**Figure 2:**
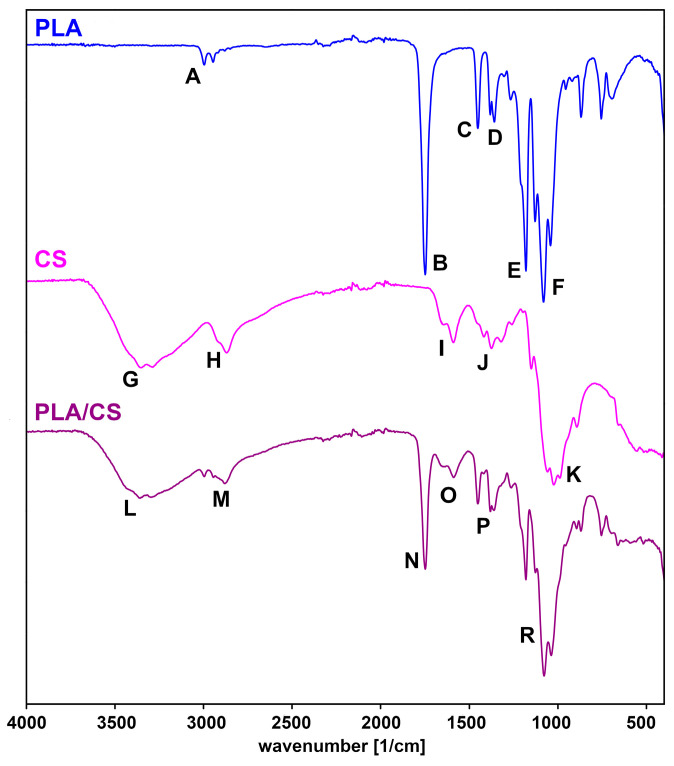
IR spectra of polylactide (PLA), chitosan (CS) and a PLA/CS scaffold.

**Figure 3:**
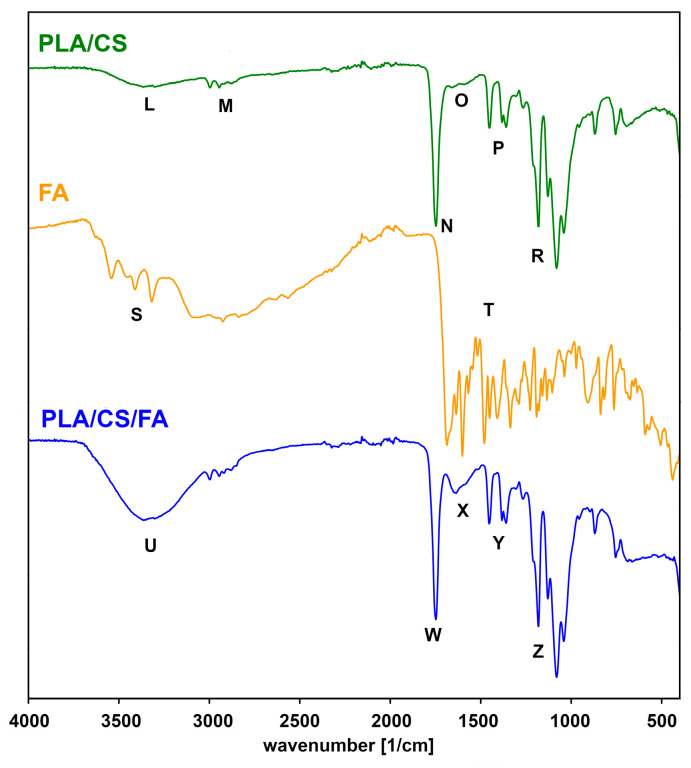
IR spectra of PLA/CS scaffold, folic acid (FA) and a PLA/CS/FA scaffold.

Additionally, infrared spectroscopy analysis (FTIR) was carried out to confirm the presence of chitosan and folic acid in the samples. For this purpose, the spectra before and after modification were compared with the spectra of the coating substances (Figure 2 and Figure 3). An interpretation was made of the most important bands present in the spectra shown.

When analysing the spectrum of the PLA, several characteristic bands can be distinguished:

- A—a band around 3000 cm^−1^ corresponds to the stretching vibrations of the C–H bonds of the CH_3_ groups;
- B—a clear high-intensity band of about 1800 cm^−1^ corresponds to the stretching vibrations of the C=O bonds;
- C—a band approx. 1400 cm^−1^ corresponds to deformation vibrations of C–H bonds;
- D—a band about 1300 cm^−1^ corresponds to deformation vibrations of C–H bonds;
- E—a band approx. 1200 cm^−1^ corresponds to tensile vibrations of C–O bonds;
- F—a band of about 1100 cm^−1^ corresponds to the stretching vibrations of the C–O bonds.

In the spectrum of chitosan, the following characteristic signals can be observed:

- G—a broad band around 3600–3000 cm^−1^ corresponds to the stretching vibrations of the O–H and N–H bonds; band broadening is due to the presence of hydrogen bonds;
- H—a band around 2900 cm^−1^ corresponds to the stretching vibrations of C–H aliphatic bonds;
- I—a band about 1600 cm^−1^ corresponds to stretching vibrations of C=O bonds and deformation vibrations of N–H bonds;
- J—a band ca. 1300 cm^−1^ corresponds to deformation vibrations of C–H bonds of CH_2_ and CH_3_ groups;
- K—a band of about 1050 cm^−1^ corresponds to the stretching vibrations of the C–O–C bonds in the glycosidic bond.

The spectrum of chitosan-modified scaffold shows signals from both polylactide and chitosan. A broad L band of about 3600–3000 cm^−1^ appears, corresponding to the stretching vibrations of the O–H and N–H bonds in chitosan, while the M band corresponds to H and A. The appearance of an O band can also be seen, which corresponds to the I band, i.e., the deformation vibrations of the N–H bonds. P and R bands result from overlapping bands D, E, F, J and K from the used substrates. It confirms the presence of chitosan in the modified scaffolds.

Figure 3 shows a comparison between the spectra of the PLA/CS scaffolds before and after the folic acid modification. The spectrum of folic acid shows two large groups of bands:

- S—a vast band around 3600–2200 cm^−1^ of overlapping vibrations originating from –NH, –OH and –CH bonds; the broadening of the band is due to the presence of numerous hydrogen bonds;
- T—an area around 1650–800 cm^−1^ of overlapping stretching and deformation bands of –C=O, –C–O, –N–H, –C–H bonds.

The PLA/CS/FA spectrum shows both folic acid and PLA/CS signals. There is an overlap between the L and S bands, resulting in a broad U band. The X band, which appeared somewhat flattened in the PLA/CS scaffold spectrum, is of higher intensity. It confirms the presence of folic acid in the modified scaffold. Some of the bands can also originate from water-bound to hydrophilic coatings.

#### 2.2.2. Interior Morphology

**Figure 4:**
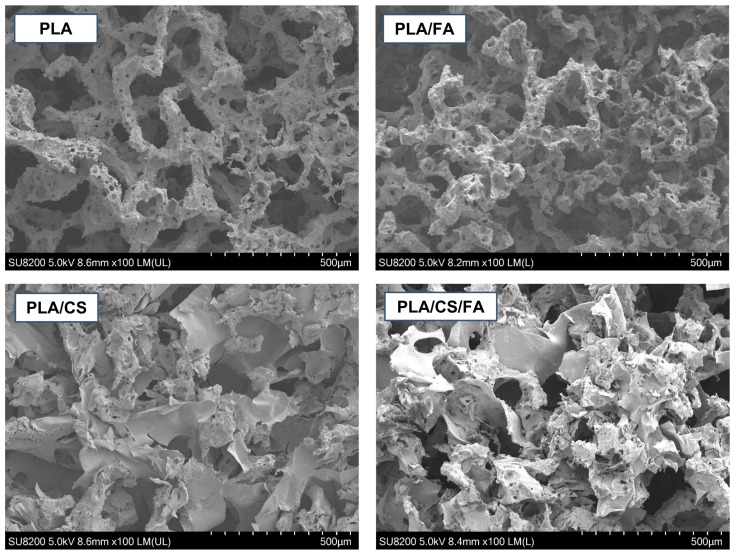
SEM images of the scaffolds cross-section.

Using scanning electron microscopy (SEM), the morphology of the obtained scaffolds was analysed. Images of the interior were taken for PLA, PLA/CS, PLA/FA and PLA/CS/FA specimens (Figure 4).

**Figure 5:**
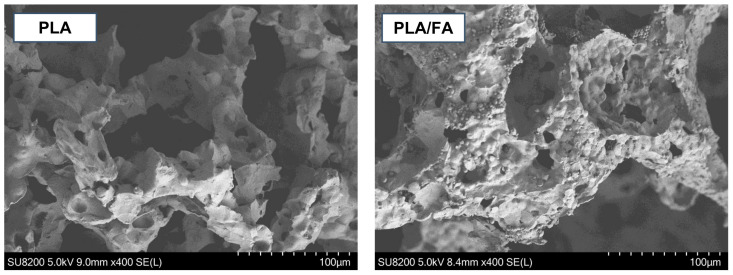
SEM images of the scaffolds before and after FA modification; higher magnification.

Polylactide scaffolds are characterised by high open porosity. The surface is highly rough and textured, with visible micropores. This structure should promote cell adhesion (providing a number of attachment sites) and allow the cell migration towards the scaffold interior, as well as the undisturbed transport of nutrients and metabolites. After modification, embedded chitosan in a flake-like form can be seen on the surface of the substitutes (compare PLA and PLA/CS). The chitosan penetrates into the pores of the scaffold, partially sealing them. However, the scaffolds retain a high open porosity and roughness of the surface. It can be seen that, despite the high viscosity of the solution, it was possible to incorporate chitosan into the structure with high efficiency. However, there is not much difference to be noticed between PLA and PLA/FA specimens, which may result from relatively small amounts of embedded folic acid. In order to better visualise possible changes in the cross-sectional surface area, those two samples were additionally compared at higher magnification (Figure 5).

Comparing the images in Figure 5, one can see the change in the surface roughness. After modification, the additional fine globular-shaped layer is visible, which may be identified as a folic acid layer.

The most important conclusion drawn from the analysis of the SEM images is that, despite the two-step modification of the scaffolds, the pore-sealing effect is not significant, and, at the same time, an increase in surface roughness is obtained. This should be an essential advantage when conducting cell cultures on the scaffolds. Introducing an additional coating to the surface and increasing its roughness provides more attachment sites for proteins and adherent cells, as was already proven in previous research in the field [24,25,26].

#### 2.2.3. Open Porosity and Mass Absorbability

**Figure 6:**
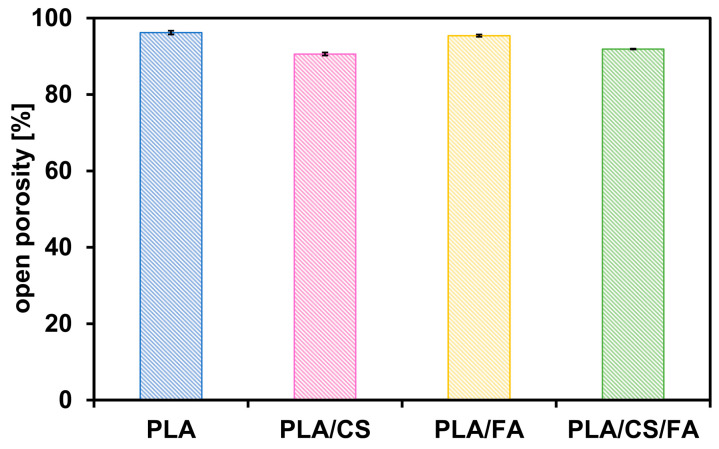
Open porosity values of the scaffolds. Error bars represent the standard deviation values.

**Figure 7:**
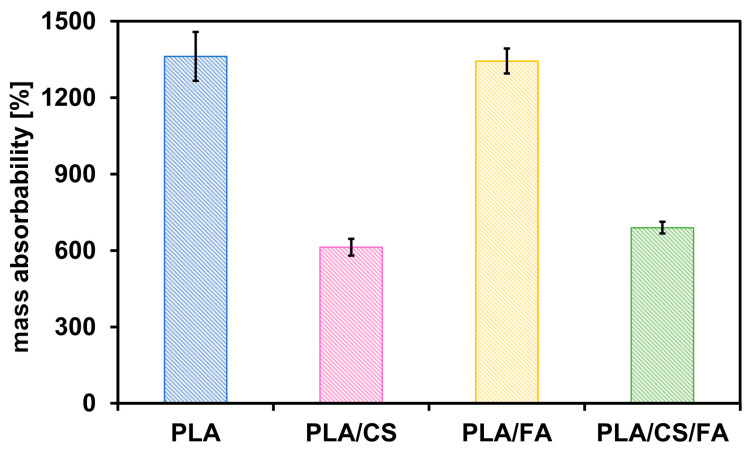
Mass absorbability values of the scaffolds. Error bars represent the standard deviation values.

Open porosity and mass absorbability are important parameters determining scaffolds’ subsequent use in tissue engineering. Cellular scaffolds should have an open porosity, which allows them to be populated with cells over their entire volume and ensures the free flow of nutrients and metabolic products. Mass absorbability determines the ability of the implant to absorb biological fluids (e.g., platelet-rich plasma to form a hydrogel-like structure) prior to placement in the patient’s body. Open porosity was, to some extent, determined by SEM imagining. Yet, it should be noted that microscopy techniques enable the determination of the small sample, not the scaffold as a whole. Therefore, open porosity was additionally determined by hydrostatic weighing, along with mass absorbability. Results are presented in Table 2 and Figure 6 and Figure 7.

Non-modified polylactide scaffolds have the highest open porosity, with a value of approximately 96%. After modification with chitosan, the open porosity is slightly reduced but still takes values above 90%. The reduction in open porosity is a result of the chitosan settling inside the pores of the scaffold and partially sealing them.

The PLA/FA scaffolds have high open porosity, similar to that of PLA scaffolds, due to the deposition of a small amount of FA, which occupied the scaffold pores to a negligible extent. In the case of the PLA/CS scaffolds, the open porosity values increased slightly after the second modification step (PLA/CS/FA), which may indicate partial leaching of the embedded chitosan. All scaffolds exhibit high open porosity, which is desirable for bone tissue engineering.

Mass absorbability varies similarly to open porosity. Non-modified scaffolds have the highest mass absorbability value. This value decreases for scaffolds modified with chitosan. As in the case of open porosity, these differences are due to the amount of chitosan introduced, which, by locating inside the scaffold, can seal its pores. The open space possible to be filled by the liquid is then reduced. For PLA and PLA/FA scaffolds, the mass absorbability values are similar. The mass absorbability of PLA/CS scaffolds increases after modification with folic acid.

#### 2.2.4. Mechanical Testing—Compressive Strength

**Figure 8:**
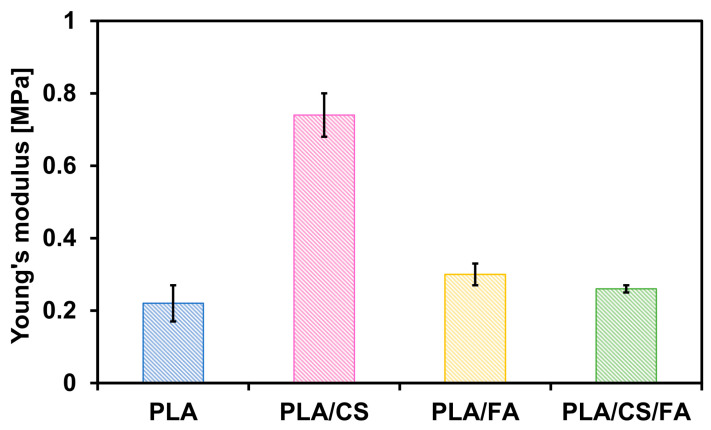
Young’s modulus values of the scaffolds. Error bars represent the standard deviation values.

The mechanical properties of the scaffolds were investigated by static compression test. Young’s modulus values were obtained, determining the elasticity of the material. The results of the analysis are shown in Table 3 and Figure 8.

When the scaffold is coated with chitosan, the value of Young’s modulus increases visibly due to the reduction of free space in the specimen and an increase in the structure’s stiffness. The addition of folic acid does not result in a significant change compared to the PLA scaffold alone. What is interesting, though, is the evident increase in the elasticity of the sample after the introduction of an FA layer over the CS coating. Apparently, the second modification step causes significant changes in the positioning of the chitosan layer in the scaffold, disrupting its stiffness.

Average Young’s moduli of cancellous bone vary substantially between 0.8 and 16.9 GPa, depending on the anatomical site, gender, age and state of health [27]. These values are significantly higher than those of the presented scaffolds. However, those polymer bone substitutes are not designed for the same applications as metal implants, intended to stabilise/support bone and provide a functional replacement. The role of such a polymeric implant is to fill minor bone defects that result from, for example, surgery. Such fillings are not designed to withstand mechanical stress, as they are intended to provide a temporary artificial matrix for the proliferation of bone cells and the restoration of natural bone at the site of the defect.

### 2.3. Cytotoxicity Testing

Cytotoxicity tests were performed to determine whether the scaffolds contained any cell toxicants. Tests were performed on extracts of the materials. Potential cytotoxic residues should be washed from the scaffolds into the culture medium.

The main cause of cell death could be residual solvents (1,4-dioxane, methanol), acetic acid or sodium bicarbonate, as both polylactide and chitosan are substances of proven biocompatibility. Another factor would be the leaching of folic acid from the coating and the acidification of the culture medium.

**Figure 9:**
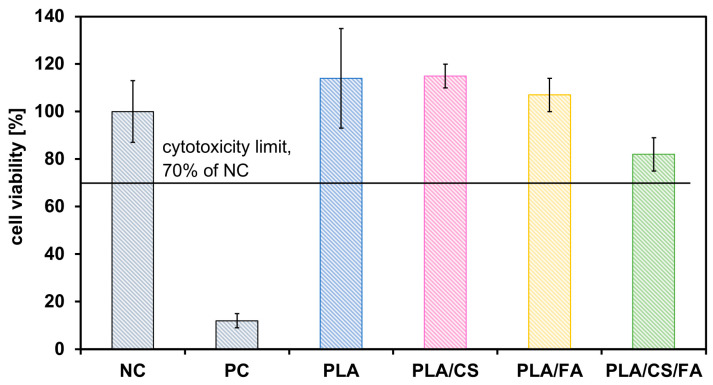
Results of cytotoxicity testing of the scaffolds: NC—negative control; PC—positive control. Cytotoxicity limit (70% of negative control) is marked by a black line. Error bars represent the standard deviation values.

The results of the cytotoxicity tests are presented in Figure 9.

All the scaffolds are non-cytotoxic in terms of possible toxic residue leaching. The tests were performed correctly, as evidenced by the difference in cell survival rates in NC and PC. Cell survival rates of approximately 100% within the standard deviation were obtained for most materials. Clearly, the lowest result was obtained for the PLA/CS/FA scaffolds. These scaffolds contained the highest amount of folic acid, which may have been washed out from within the scaffold, as also evidenced by the yellow colouration of the extract. Although the folic acid coating does not acidify the culture medium, too high a concentration in the extract may have acted negatively on the cells and reduced their viability. More precise conclusions on the effect of chitosan coatings and folic acid on cell adhesion and proliferation will be possible in the future by performing cell cultures on the scaffolds.

## 3. Conclusions

The result of the conducted research is the development of a new procedure for modifying polylactide substitutes using folic acid and the application of this procedure in combination with chitosan modification. The described studies confirm the effectiveness of the modifications with chitosan and folic acid to obtain gel-like biomimetic coatings. The viscosity of the chitosan used was found to affect the effectiveness of the modification. Folic acid binds to the chitosan present on the surface of the scaffolds.

The introduction of biomimetic coatings on the surface of the scaffolds increases their mechanical strength while having no adverse effect on their morphology. The modified substitutes are characterised by high values of open porosity and mass absorbability. The open porosity test confirmed that the resulting scaffolds have more than 90% open porosity. This allows the free flow of nutrients to the interior and metabolites to the exterior of the scaffold. The scaffolds also had high mass absorbability values, translating into the ability to be saturated with biological fluids before implantation into the patient’s body.

The substitutes before and after modification have a lower Young’s modulus than spongy bone, but this is not a disadvantage. These types of polymeric implants are intended for filling minor bone defects arising, for example, during bone reconstruction procedures. The purpose of such fillings is not to bear mechanical loads, whereas the greater flexibility of these substitutes will facilitate their adaptation to the patient’s bone defect.

To examine the cytotoxic effect, tests were performed on extracts of the materials using cells of the L929 line. The scaffolds were shown to be non-cytotoxic, i.e., they did not leach toxic residues that could cause cell death.

All of the obtained scaffolds meet the initial requirements for implants intended for bone regeneration.

## 4. Materials and Methods

### 4.1. Polylactide Scaffolds Preparation

A 3% *w*/*w* solution of poly-L-lactide in 1,4-dioxane was prepared. The solution was stirred for 24 h at room temperature. Then, the solution was heated in a water bath to 60 °C and stirred at approximately 600 min^−1^. Using a syringe, a porophore (MiliQ water) was added dropwise (at a ratio of 1.5 mL water per 20 mL of PLLA solution). The solution was cooled to room temperature and left for 24 h. Next, the solution was poured into cylindrical moulds, with each receiving 20 mL, and frozen at −18 °C for 24 h. Frozen samples were removed from the moulds, placed in 300 mL of cold methanol bath and left at −18 °C for five days. After that, the samples were placed individually in beakers with 300 mL of distilled water and stirred at a rate of 120 min^−1^ for 24 h, with water exchanged twice, after 3 and 6 h. The scaffolds were then vacuum-dried at 40 °C under 10 mbar for 24 h.

### 4.2. Modification of Scaffolds with Chitosan

A 2% *w*/*v* chitosan solution in a 1% *w*/*w* solution of acetic acid in water was prepared. Chitosan was added in portions to the acetic acid solution. A scaffold was placed in a vacuum ampoule and weighted down with a stirring element. A total of 20 mL of a chitosan solution was poured into the ampoule. Immersion under reduced pressure was carried out for 30 min. After this, the substitute was wrapped in parafilm, placed on a Petri dish and frozen for 24 h at −18 °C. The scaffolds were then dried in a vacuum chamber for 48 h at room temperature.

A solution of sodium bicarbonate in a water–methanol mixture (volume ratio 3:1) was prepared with a concentration of 1.16% *w*/*v*. A chitosan-modified scaffold was placed in a vacuum ampoule and weighted with a stirring element. Then, 25 mL of a sodium bicarbonate solution was poured into the ampoule. The sodium bicarbonate solution was immersed under reduced pressure for 10 min. The scaffold was transferred to a beaker with 150 mL of distilled water and placed on a magnetic stirrer (temperature 37 °C, stirring speed 120 min^−1^) for 3 h. The water was changed twice—after 1 and 2 h. After this time, the scaffolds were vacuum dried at 40 °C under 10 mbar for 48 h.

### 4.3. Modification of Scaffolds with Folic Acid

A 100 mL of 8% (*w*/*v*) solution of sodium bicarbonate in water was prepared, and subsequently, 5 g of folic acid was added. The process was carried out at room temperature. The solution was used to modify the scaffolds with and without chitosan coating. Soaking in the folic acid solution was carried out for 30 min under reduced pressure. After this time, the scaffolds were rinsed in an acetic acid solution in water (pH = 6), then wrapped in parafilm and frozen for 24 h at −18 °C. The scaffolds were vacuum-dried at 40 °C for 48 h.

### 4.4. Fourier-Transform Infrared Spectroscopy (FTIR)

Small sections were cut from each scaffold type and examined by Fourier-transform infrared spectroscopy. IR spectra were taken with Bruker’s ALPHA spectrometer with ATR attachment.

### 4.5. Elemental Analysis

Small sections of the interior of each type of scaffold weighing approximately 100 mg were taken. Elemental analysis was carried out using an Elementar Vario EL III apparatus.

### 4.6. Scanning Electron Microscopy (SEM)

Small sections of the inner surface of the scaffolds were taken. Images were taken using a Hitachi SU 8230 ultra-high-resolution scanning-transmission electron microscope provided by the Centre for Advanced Materials and Technology CEZAMAT, Warsaw University of Technology.

### 4.7. Hydrostatic Weighing

Open porosity (P_o_) and mass absorbability (A_m_) were determined by hydrostatic weighing. A Mettler Toledo XS 104 balance was used. In the first step, the dry scaffolds were weighed in air (m_1_). The scaffolds were then soaked in isopropanol under reduced pressure. In the next step, the wet scaffolds were weighed in (m_2_) and then out (m_3_) of the reference liquid.

The values of open porosity and mass absorbability were determined according to the following formulas:

(1) $$P_{o}=\frac{m_{3}-m_{1}}{m_{3}-m_{2}}\cdot 100\%$$

(2) $$A_{m}=\frac{m_{3}-m_{1}}{m_{1}}\cdot 100\%$$

Each sample was tested in triplicate, and the mean and standard deviation were determined.

### 4.8. Static Compression Test

A cylinder approximately 6–7 mm high was cut from each specimen. The test was performed on a Tinius Olsen H104S apparatus using QMat 5M5 software with a 250 N strain gauge. The compression rate was 10% of the specimen height/min. Each sample was tested in triplicate, and the mean and standard deviation were determined.

### 4.9. Cytotoxicity Tests on Extracts

All procedures were carried out in a UV-sterilised laminar flow cabinet. To perform the cytotoxicity tests, mouse fibroblast line L929 (ATCC) was used. All culture media have been supplied by Gibco, including 1 g/L Dulbecco’s Modified Eagle Medium (DMEM) supplemented with 10% *v*/*v* inactivated fetal bovine serum and 1% *v*/*v* Pen-Strep.

#### 4.9.1. Maintaining L929 Cells

The used culture medium was aseptically pipetted out from culture flasks, and the remaining cell monolayer was rinsed twice with sterile DPBS without calcium and magnesium, 3 mL of sterile 0.05% trypsin-EDTA was poured inside, and the flasks were incubated for 5 min at 37 °C. Suspension of detached cells was transferred to a sterile Falcon-type centrifuge tube, 10 mL of fresh culture medium was added, and the Falcon-type tube was centrifuged at 4500 min^−1^ for 5 min. The supernatant was aseptically removed, and the cells were suspended in a 10 mL fresh culture medium. Cells were passaged into new culture flasks and incubated at 37 °C in a 5% CO_2_-enriched air atmosphere.

#### 4.9.2. XTT Cytotoxicity Test

Sample discs of 1.7 cm diameter were placed in 24-well plates, vacuum-packed and sterilised. Extracts were made by pouring 2.0 mL of culture medium into each well. The plates were incubated at 37 °C for 24 h.

A cell suspension of 10^5^ cells/mL was placed in a 96-well plate, 100 μL per well. The outer wells were flooded with DPBS to prevent evaporation of the culture medium. The plates were incubated at 37 °C for 24 h. After removing the medium, the extracts were added, 100 μL per well. Negative control wells were flooded with fresh medium, and positive control wells were flooded with 0.1% solution of the Triton X-100 in DMEM. The plate was incubated at 37 °C for 24 h in a 5% CO_2_-enriched air atmosphere.

The used culture medium was removed, and the cells in the 96-well plate were washed twice with 100 μL of DPBS. A 33% *v*/*v* solution of XTT reagent (CyQUANT XTT Cell Viability Assay, Thermo Fisher Scientific, Waltham, Massachusetts, U.S.) in culture medium was added, 150 μL per well. The 96-well plate was placed in an incubator for 4 h at 37 °C. The absorbance was measured using the multiwell plate reader for 450 and 630 nm. The results were averaged and compared to the negative control, counted as 100% of the metabolic activity of cells.

## Figures and Tables

**Table 1:** Comparison of the chemical composition of modified and non-modified scaffolds.

Scaffold Type	% N	% C	% H	% O
PLA	-	49.95 ± 0.09	5.20 ± 0.13	44.85 ± 0.04
PLA/CS	2.59 ± 0.01	46.81 ± 0.04	5.34 ± 0.07	45.26 ± 0.02
PLA/FA	0.75 ± 0.04	50.00 ± 0.11	4.55 ± 0.16	44.70 ± 0.09
PLA/CS/FA	4.44 ± 0.16	39.64 ± 0.19	4.56 ± 0.16	51.36 ± 0.19

**Table 2:** Scaffold open porosity and mass absorbability values.

Scaffold Type	Open Porosity [%]	Mass Absorbability [%]
PLA	96.2 ± 0.5	1360 ± 100
PLA/CS	90.6 ± 0.4	610 ± 30
PLA/FA	95.4 ± 0.1	1340 ± 20
PLA/CS/FA	91.9 ± 0.1	690 ± 10

**Table 3:** Young’s modulus values of the scaffolds.

Scaffold Type	Young’s Modulus [MPa]
PLA	0.22 ± 0.05
PLA/CS	0.74 ± 0.06
PLA/FA	0.30 ± 0.03
PLA/CS/FA	0.26 ± 0.01

## Data Availability

The data presented in this study are available on request from the corresponding author.
